# Polymorphisms in immunoregulatory genes and the risk of histologic chorioamnionitis in Caucasoid women: a case control study

**DOI:** 10.1186/1471-2393-5-4

**Published:** 2005-02-21

**Authors:** Margaret F Annells, Prue H Hart, Charles G Mullighan, Susan L Heatley, Jeffrey S Robinson, Helen M McDonald

**Affiliations:** 1 Microbiology and Infectious Diseases, Women's and Children's Hospital, 72 King William Road, North Adelaide, South Australia, 5006, Australia; 2 Microbiology and Infectious Diseases, Flinders University, Bedford Park, South Australia, 5042 Australia; 3 Australian Red Cross Blood Services, South Australian Branch, 301 Pirie Street Adelaide, South Australia, 5000, Australia; 4 Obstetrics and Gynaecology, University of Adelaide, 5005, Australia

## Abstract

**Background:**

Chorioamnionitis is a common underlying cause of preterm birth (PTB). It is hypothesised that polymorphisms in immunoregulatory genes influence the host response to infection and subsequent preterm birth. The relationship between histologic chorioamnionitis and 22 single nucleotide polymorphisms in 11 immunoregulatory genes was examined in a case-control study.

**Methods:**

Placentas of 181 Caucasoid women with spontaneous PTB prior to 35 weeks were examined for histologic chorioamnionitis. Polymorphisms in genes *IL1A*, *IL1B*, *IL1RN*, *IL1R1*, tumour necrosis factor (*TNF*), *IL4*, *IL6*, *IL10*, transforming growth factor beta-1 (*TGFB1*), Fas (*TNFRSF6*), and mannose-binding lectin (*MBL2*) were genotyped by polymerase chain reaction and sequence specific primers. Multivariable logistic regression including demographic and genetic variables and Kaplan-Meier survival analyses of genotype frequencies and pregnancy outcome were performed.

**Results:**

Sixty-nine (34%) women had histologic evidence of acute chorioamnionitis. Carriage of the *IL10*-1082A/-819T/592A (*ATA*) haplotype [Multivariable Odds ratio (MOR) 1.9, P = 0.05] and *MBL2 *codon 54Asp allele (MOR 2.0, P = 0.04), were positively associated with chorioamnionitis, while the *TNFRSF6*-1377A/-670G (*AG*) haplotype (MOR 0.4, P = 0.03) and homozygosity for *TGFB1*-800G/509T (*GT*) haplotype (MOR 0.2, P = 0.04) were negatively associated.

**Conclusion:**

These findings demonstrate that polymorphisms in immunoregulatory genes *IL10*, *MBL2*, *TNFRSF6 *and *TGFB1 *may influence susceptibility to chorioamnionitis.

## Background

Acute infection of the amniotic fluid and chorioamnion is a common cause of preterm delivery before 34 weeks gestation. Consistent with previous reports [1], an Australian study found that 31% of preterm births (PTB) before 34 weeks had histologic chorioamnionitis [2]. An inverse relationship between delivery gestation and histologic chorioamnionitis was shown with histologic chorioamnionitis identified in 66% of births before 25 weeks. Chorioamnionitis usually results from ascent of pathogenic organisms such as Group B streptococcus and *Escherichia coli *[3], from the lower genital tract. A T helper cell type1 (Th1) cytokine host response occurs with the production of pro-inflammatory cytokines such as (IL-1β and TNF) produced mainly by activated monocytes/macrophages in amnion, chorionic and decidual tissue. The increased levels of these cytokines trigger prostaglandin F2 and E2 biosynthesis by the decidua and amnion, inducing uterine contractions and labour [4]. In the setting of infection, IL-1 and TNF have a synergistic effect on the acute phase cytokine IL-6 with increased production in trophoblasts and chorion and to a lesser extent decidua [5]. The intensity and duration of the inflammatory response is modulated by T helper cell type 2 cytokines and anti-inflammatory mediators such as IL-4, IL-10, IL-1 receptor antagonist (IL-1ra) and transforming growth factor beta-1 (TGFβ-1).

The innate host defense system is also an important factor in pregnancy and the body's response to infection. Mannose-binding lectin (MBL), a protein of the complement system, opsonises pathogens independently of antibody for phagocytosis and influences inflammatory pathways [6]. There are several common polymorphisms in the promoter and coding region of the *MBL2 *gene that profoundly influence circulating levels of functional, multimeric MBL. These genetic variants and low MBL levels are associated with risk and severity of infection in a variety of clinical contexts [6].

Fas, a cell surface receptor of the TNF/nerve growth factor superfamily, mediates apoptosis after binding Fas ligand [7]. Activated leucocyte populations, including T cells, express high amounts of Fas and evidence suggests that trophoblast Fas-mediated apoptosis is increased in women with chorioamnionitis [8].

The immune system operates as a complex regulatory network of cytokines and other mediators with a degree of redundancy that changes as pregnancy progresses [9]. This makes it difficult to assess the importance of single cytokines in inflammation and response to infectious agents when examined in isolation. The genes encoding these mediators contain coding and non-coding polymorphisms that influence level or function of the encoded mediators [10]. These polymorphisms may influence susceptibility to chorioamnionitis and adverse pregnancy outcome. Single nucleotide polymorphisms (SNPs) for *IL1 *+3953 and *TNF *-308 are associated with human chorioamnionitis in *in vitro *studies [11] and carriage of the *TNF *-308 A allele has been reported as a risk factor for clinical chorioamnionitis in pregnant women [12]. However these reports were restricted to small numbers of cytokine genes and did not include the analysis of polymorphisms in molecules important in apoptosis and host defence. Examination of a range of immunoregulators and SNPs concurrently is more likely to identify which genes and alleles are of primary importance in response to intra-uterine infection.

We hypothesized that polymorphisms in immunoregulatory genes may influence susceptibility to chorioamnionitis and subsequent preterm labour. To examine this hypothesis, we investigated multiple polymorphisms in immunoregulatory genes in a population of Australian Caucasoid women experiencing spontaneous PTB before 35 weeks and histologic chorioamnionitis.

## Methods

This case-control study was approved by the Women's and Children's Hospital Ethics and Research Committee. A total of 368 unrelated women of child-bearing age who had obstetric management at the Women's and Children's Hospital (WCH) and history of PTB before 35 weeks gestation were identified from the hospital data base. All women were approached in the first instant by letter of introduction and invited to participate in the study. For women living in the Adelaide metropolitan area, the study nurse made a home visit to those who volunteered for eligibility assessment. Geographical distance excluded 50, 66 did not respond, 40 were not eligible and 31 declined. Of the women enrolled some had more than one PTB. The PTB fitting the study criteria and closest to the enrolment date became the index birth. Written consent was obtained and a 9 ml peripheral blood sample was collected for isolation of leucocytes and DNA extraction. 181 women with a history of spontaneous preterm labour and subsequent PTB between 20 and 35 weeks in the index pregnancy had histological examination of the placenta performed. Histopathology staff members were unaware of the genetic results.

Clinical data recorded included gestational age at delivery (calculated by ultrasonography at approximately 18 weeks). Obstetric factors known to place a woman at risk of preterm labour or preterm prelabour rupture of membranes (PPROM) (defined as prelabour rupture of membranes 3 or more hours before preterm delivery) were also recorded. Placental examination and swabbing was performed by staff histopathologists [13] and included examination of the placental tissue in blocks, extra-placental membranes in a "roll" (amnion, chorion and decidua) and the umbilical cord in cross section. Chorioamnionitis was defined as a dense polymorphonuclear leukocyte infiltration of the chorionamniotic component of the placenta and membranes (amnion, chorion, amniochorial membranes and/or chorionic plate (top part of the fetal side of the placenta). Inflammatory reaction confined to either the decidua or subchorial intervillous space of the placental disc, without infiltration of the chorion or amnion was recorded as negative chorioamnionitis [3]. Funisitis was defined as inflammation in one or more of the umbilical cord vessels (vasculitis) with or without inflammation in the Wharton's jelly (the supporting soft tissue around the vessel in the cord). The definition of acute chorioamnionitis included funisitis and/or amnionitis. In both groups a number of women had PPROM, occasional bleeding during pregnancy, recorded clinical chorioamnionitis or a history of prior term births. Tocolytic therapy and intravenous antibiotics were used according to obstetric protocols. All participants were Caucasoid, unrelated and at least 18 years of age at enrolment. Women with diagnosed autoimmune disease, pre-eclampsia, in vitro fertilisation treatment, confirmed uterine malformations, no spontaneous labour, ultrasound-confirmed fetal abnormality, insulin dependent diabetes, or multiple pregnancy were excluded.

Demographic characteristics, a comprehensive medical and obstetric history, age, any smoking, alcohol consumption, substance use and clinical chorioamnionitis (defined as any three of the following – white blood cell >1500 × 10^9/L, three consecutive C reactive protein readings ≥ 15mg/L, pyrexia ≥ 38°C, uterine pain or tenderness) were verified from case records. Clinical chorioamnionitis was not used as a subgroup for polymorphic analysis because this did not include women with sub-clinical infection. Since the risk of chorioamnionitis is not as great in PTB after 35 weeks, women with PTB between 35 and 37 weeks were not enrolled [2].

Genes were chosen if reported to be associated with inflammatory disease[10] and if existing functional data suggested a role in chorioamnionitis and preterm delivery[5,11]. SNPs were selected where the minor allele has a frequency >10%. Several genes were known to contain polymorphisms at multiple sites that are of functional importance (e.g. the 5'and coding variants of *MBL2*) [14], thus multiple variants were genotyped and the haplotype recorded. SNPs in the IL1 gene cluster, *TNF*, *IL4*, *IL6*, *IL10*, *TGFB1*, *TNFRSF6*, and *MBL2 *genes and their relationship with histologic chorioamnionitis were therefore examined in a cohort of women with spontaneous PTB.

Peripheral blood samples were blinded and tested at the Australian Red Cross Blood Service, Adelaide (ARCBS). Genomic DNA was extracted from ethylenediaminetetraacetic acid anticoagulated venous blood using standard methods as previously described [15]. SNPs for *TNF *(+488,-238,-308) [16], *IL1A *(-889), *IL1B *(+3962, -511), *IL4 *(-590), *IL10 *(-1082, -819, -592), *TGFB1 *(-800, -509) [17], *MBL2 *[-550, -221, codon 52 (Arg → Cys), codon 54 (Gly → Asp), codon 57 (Gly → Glu)] [18], *IL6 *(-174), IL-1 receptor antagonist *IL1RN *(+11100), IL-1 type 1 receptor *IL1R1 *(+970) and *TNFRSF6 *(-1377, -670) [19] were genotyped using the polymerase chain reaction and sequence specific primers (PCR-SSP). Multiple polymorphisms were examined in the *IL10*, *TNFRSF6*, *TNF*, *TGFB1 *and *MBL2 *genes by PCR haplotyping. The term "haplotype" denotes ordered combination of alleles on a single chromosome, and is used where multiple polymorphisms are genotyped by PCR-haplotyping for a single gene. For PCR haplotyping combinations of forward and reverse allele-specific-primers were used to directly amplify alleles on the same chromosome [20]. This technique also provides a degree of redundancy, in that multiple reactions genotype a polymorphism, thus confirming results. All PCR-SSP reactions have been extensively used in the same laboratory. Internal control primers for conserved regions of the *DRB1 *(major histocompatibility complex, class 11 DR beta 1) and *APC *(adenomatous polyposis coli) genes were included in every PCR mix to verify successful amplification. All genes except the *IL1A *had a repeat genotyping rate of less than 1%. The *IL1A *had a repeat rate of 10%.

The sample size was calculated by using the statistical software package EpiInfo 6 (Version 6.04d, Centre for Disease Control and Prevention, Atlanta, GA). We estimated the study had sufficient power (1-β = 80%, P = 0.05) to detect a minimum of 17% difference in genotype frequency between chorioamnionitis and no chorioamnionitis for frequencies of approximately 10% (ie. 10% versus 27%). For a genotype frequency of 3% the minimum detectable difference was 10% (3% versus 13%). Allele and haplotype frequencies for polymorphisms were determined by direct counting from each chromosome. Frequencies of genetic variants were recorded as allele (proportion of positive chromosomes), genotype (proportion possessing homozygous or heterozygous combinations of alleles), and 'carriage' of an allele or haplotype (either in homozygous or heterozygous state). Alleles were said to be in Hardy-Weinberg equilibrium if the observed genotype frequencies did not differ significantly (P > 0.05) from those expected when analysed by Chi square. Gene variant frequencies were compared using 2 × 2 contingency table analysis and the Chi square test for independence using EpiInfo 6. Mean birth weights were compared by *t*-test.

Multivariable analysis was performed by stepwise backwards multiple logistic regression using SYSTAT 9.0 software (Systat version 1999 SPSS Inc). The basic model included genetic variables with P values < 0.15 obtained in univariate analyses. In each multivariable model, only one genotype or haplotype of each gene was selected for analysis. Selection of genotype/haplotype for the model was based on strength of association, the previously reported importance of a gene variant and homozygosity of alleles. At each backward step in the regression analysis P values ≥ 0.07 were rejected. Kaplan-Meier survival analysis (SYSTAT 9.0) was used to identify cumulative probability in maintaining pregnancy by gestation stratified by chorioamnionitis and gene variables. Breslow-Gehan Chi square was used to weight each gestational period by the total number at risk at that time so that earlier gestations receive greater weight than later gestations.

## Results

Sixty-nine (38%) women had histologic evidence of acute chorioamnionitis, and 112 (62%) had no such evidence. There were no significant differences between those with and without histologic chorioamnionitis for maternal age, race, history of smoking, alcohol consumption, substance use, new partner, cervical cerclage, placenta praevia after 22 weeks with bleeding, history of two or more PTBs before 35 weeks, miscarriage between 12 and 20 weeks or PPROM (Table 1). Histologic chorioamnionitis was positively associated with clinical chorioamnionitis [Univariate Odds Ratio (OR) 6.1, 95% Confidence Intervals (CI) 2.4–17, uncorrected P ≤ 0.0001], PTB before 29 weeks (OR 7.6, CI 3.4–18, P ≤ 0.0001) and positive placental culture (OR 3.0, CI 0.9–11, P = 0.04).

**Table 1:** Demographic and clinical characteristics of women with preterm birth before 35 weeks.

**Variable**	**Histologic Chorio-amnionitis n = 69**	**No Histologic Chorio-amnionitis n = 112**	**Significant OR (95% CI), and Uncorrected P values**
Maternal age (years)	29 (16–41)	30 (17–44)	
Delivery gestation (weeks)	28 (20–34)	30 (23–34)	*
Mean birth weight	1399 (± 676)	1897 (± 504)	< 0.0001
Maternal age ≥ 35 years	11 (16%)	18 (16%)	ns
Maternal age ≤ 17 years	2 (3%)	2 (2%)	ns
Any smoking	22 (31%)	29 (26%)	ns
Any alcohol	44 (64%)	73 (65%)	ns
Any substance use	7 (10%)	10 (9%)	ns
Partner change for	14 (20%)	20 (18%)	ns
pregnancy			
Cervical cerclage	3 (4%)	2 (2%)	ns
Previous mid-trimester	4 (6%)	12 (11%)	ns
miscarriage (12–20 weeks)			
2 or more preterm births	11 (16%)	20 (18%)	ns
Placenta praevia after 22 weeks with bleeding	1 (1%)	2 (2%)	ns
Documented clinical chorioamnionitis	22 (32%)	8 (7%)	6.1 (2.4–17), < 0.001
PPROM ≥ 3 hours	39 (57%)	74 (66%)	ns
Gestation < 29 weeks	33 (48%)	12 (11%)	7.6 (3.4–18), < 0.001
Documented positive			
placental culture	10 (14%)	6 (5%)	3.0 (0.9–11), 0.04
Neonatal sepsis	10 (14%)	0	19 (3 – 823), < 0.001
Neonatal death	6 (9%)	3 (3%)	ns
Post partum endometritis	5 (7%)	3 (3%)	ns

Descriptive statistical results are mean (± SD) or number (%) as appropriate, and maternal age and gestation as median (range). Characteristics are from index birth. PPROM = Preterm premature rupture of membranes 3 hours or more before delivery < 35 weeks.

Univariate analyses were tested using Chi square analysis of 2 × 2 table, and uncorrected P values, Odds Ratio (OR), and 95% Confidence Intervals (CI), P values ≤ 0.05 are presented. Mean birth weights were tested by two-sample *t*-test, assuming unequal variances.

* See Figure 1,

ns, not significant.

All alleles were in Hardy-Weinberg equilibrium. All gene variants were tested for associations between the two groups. In preterm women without histologic chorioamnionitis frequencies for the *IL10*-1082A/-819T/-592A (*ATA*) haplotype [17], *MBL2 *codon 54Asp (the MBL 'B' allele) [21], *TNFRSF6*-1377A/-670G (*AG*) haplotype [22] and *TGFB1*-800G/-509T (*GT*) homozygosity [17] were similar to reported Caucasian controls.

In a comparison of women with chorioamnionitis and those without chorioamnionitis a comprehensive listing of gene carriage, alleles, genotype and haplotype frequencies is provided (Additional file 1). Univariate analysis revealed that carriage of the *IL10*-1082A/-819T/-592A (*ATA*) haplotype (present in 49% of women with chorioamnionitis vs 33% without chorioamnionitis, OR 2.0, P = 0.03) and the *IL10*-819T and -592A alleles (50% versus 33%, OR 2.0, P = 0.03) were positively associated with histologic chorioamnionitis, as was carriage of the *MBL2 *54Asp (B allele) (39% versus 25%, OR 1.9, P = 0.04) (Table 2). Although the frequency of the common *TNFRSF6*-1377 G/G genotype was higher in women with histologic chorioamnionitis (0.87 versus 0.73, OR 2.4, P = 0.03), the variant *TNFRSF6*-1377A/-670G (*AG*) haplotype (OR 0.4, P = 0.03) and homozygosity for the *TGFB1 *haplotype -800G/-509T (*GT*) (OR 0.2, P = 0.03) were negatively associated with chorioamnionitis (Table 2). No other significant associations with gene variant frequencies were seen in univariate analysis.

**Table 2:** Genes, alleles, haplotypes and genotypes in women with preterm birth before 35 weeks and chorioamnionitis.

**Gene**	**Histologic Chorio-amnionitis n = 69**	**No Histologic Chorio-amnionitis n = 112**	**Univariate analysis OR (95% CI) Uncorrected P value**	**Multivariable analysis OR (95% CI), P value**
***IL10***				
**Allele**-819 T	34 (0.50)	37 (0.33)	2.0 (1.0–3.8), 0.03	
-592 A	34 (0.50)	37 (0.33)	2.0 (1.0–3.8), 0.03	
**Haplotype**				
-1082A/-819T/-592A	34 (0.49)	37 (0.33)	2.0 (1.0–3.8), 0.03	1.8 (1.0–4.0), 0.05
**Genotype**				
-819 C/T	28 (0.41)	28 (0.25)	2.1 (1.0–4-1), 0.03	
-592 A/C	28 (0.41)	28 (0.25)	2.1 (1.0–4-1), 0.03	
***MBL2***				
Codon 54Asp ('B')	27 (0.39)	28 (0.25)	1.9 (1.0–3.9), 0.04	2.0 (1.0–4.0), 0.04
***TNFRSF6***				
**Allele**-1377A	9 (0.13)	30 (0.27)	0.4 (0.2–1.0), 0.03	
**Haplotype**				
-1377A/-670G	9 (0.13)	30 (0.27)	0.4 (0.2–1.0), 0.03	0.3 (0.2–0.9), 0.03
**Genotype**				
-1377 G/G	60 (0.87)	82 (0.73)	2.4 (1.0–6.3), 0.03	
***TGFB1***				
**Haplotype**				
-800G/-509T	2 (0.03)	14 (0.13)	0.2 (0.02–1.0), 0 .03	0.2 (0.04–0.9), 0.04
Homozygosity				

Data are presented as number and frequencies of alleles, genotypes and haplotypes.

Univariate analyses were tested using Chi square analysis of 2 × 2 table, and uncorrected P values, Odds Ratio (OR), and 95% Confidence Intervals (CI), P values ≤ 0.07 are presented.

Multivariable analyses included genetic variables with P values ≤ 0.15 using backward step multiple logistic regression. Multivariable model included carriage of *IL10 ATA*, *MBL2 *codon 54Asp, *TNFRS6*-1377A/-670G (*AG*) haplotype, and homozygous *TGFB1*-800G/-509T, *TNF *+488 G/A, and *IL1R1 *+970 T/T genotypes.

*MBL2 *codon 54Asp (allele 'B') is a single nucleotide polymorphism in exon 1 codon 54 (Gly/Asp).

*TNFRSF6 *is the gene symbol for Fas; ns, not significant.

For confirmation of independence of association, a multivariable model was used including univariate variables with P values ≤ 0.15. This model included the above genetic variables (Table 2) plus *TNF *+488 G/A genotype (25% versus 15%, OR 1.8, P = 0.11) and *IL1R1 *+970 T/T (6% versus 14%, OR 0.4, P = 0.08) from Additional file 1. Multivariable analysis confirmed that carriage of the variant *IL-10 ATA *haplotype [Multivariable Odds Ratio (MOR) 1.9, P = 0.05)] and *MBL2 *codon 54Asp (MOR 2.0, P = 0.04) were independently associated with histologic chorioamnionitis, while homozygosity for the variant *TGFB1*-800G/-509T (*GT*) haplotype (MOR 0.2, P = 0.04), and carriage of *TNFRSF6*-1377A/-670G (*AG*) haplotype (MOR 0.4, P = 0.03) remained negatively associated with histologic chorioamnionitis. The remainder of SNPs tested showed no significant differences.

**Figure 1:**
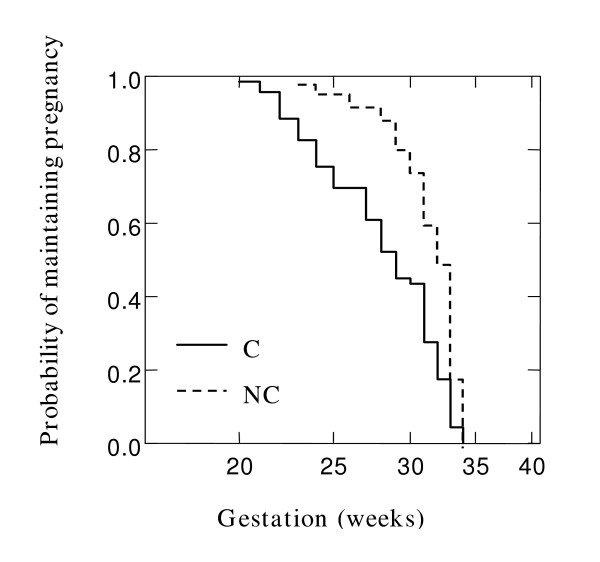
**Relationship of chorioamnionitis with gestation at delivery. **Kaplan-Meier survival estimates in 181 women where all gestations proceeded to delivery < 35 weeks. Effect of chorioamnionitis (C) versus no chorioamnionitis (NC), P < 0.001. Significance was tested using Breslow-Gehan Chi square analysis with 1 degree of freedom.

**Figure 2:**
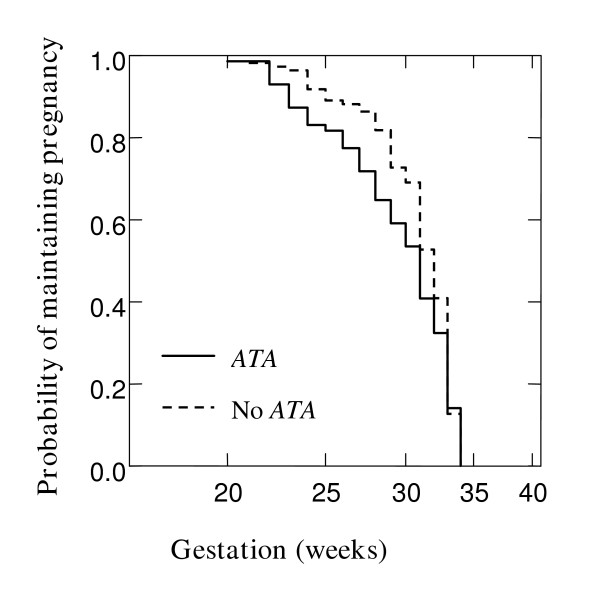
**Relationship of *IL10 ATA *haplotype with gestation at delivery. **Kaplan-Meier estimates in 181 women where all gestations proceeded to delivery before 35 weeks. Effect of *IL10 ATA *haplotype versus No *ATA*, P = 0.06. Significance was tested using Breslow-Gehan Chi square analysis with 1 degree of freedom.

To examine the cumulative probability of maintaining a pregnancy and effect of chorioamnionitis on gestation at delivery in the overall cohort, Kaplan Meier survival analysis was performed. Figure 1 shows that chorioamnionitis was more common in PTB at earlier gestations (Breslow-Gehan, Chi square 36 with 1 degree of freedom, P ≤ 0.001). In addition, the cumulative probability of maintaining a pregnancy was stratified on the carriage of *IL10 ATA *haplotype (Figure 2). Women with this *IL10 ATA *haplotype were more likely to deliver at earlier gestations (Breslow-Gehan, Chi square 3.5 with 1 degree of freedom, P = 0.06) (Figure 2). There was no evidence of the effect of *MBL2*, *TGFB1*, *or TNFRSF6 *SNPs on gestation of PTB.

## Discussion

These results extend prior reports investigating gene polymorphisms and the pathogenesis of PTB and chorioamnionitis [11]. In this study we found significant positive associations between the carriage of the *IL10 ATA *haplotype, *MBL2 *codon 54Asp and histologic chorioamnionitis, and negative associations with *TNFRSF6 *and *TGFB1*. This is the first study to investigate *TNFRSF6 *and *TGFB1 *in combination with such a wide range of cytokine SNPs in histologic acute chorioamnionitis. Also, this is one of the few studies that have used multivariable and survival analyses for investigating adverse pregnancy outcomes such as chorioamnionitis and PTB.

The differences in genotype distribution for *IL10 *and *MBL2 *may be of biologic importance in the pathogenesis of histologic chorioamnionitis. The function of IL-10 in pregnancy is of particular interest because of its role in the homeostatic control of an inflammatory immune response [23]. Although there have been conflicting reports the majority suggest that the *IL10 ATA *promoter haplotype and alleles comprising the haplotype are associated with low protein levels in Caucasians [24]. Low levels of IL-10 have been associated with PTB in rat models [25]. In a pregnant rhesus monkey model, combined intra-amniotic and maternal systemic IL-10 administration inhibited IL-1β-induced preterm uterine contraction [26]. Survival analysis showed that the *IL10 ATA *haplotype may influence the timing of PTB. IL-10 antagonises the synthesis and actions of pro-inflammatory cytokines such as IL-1 [27]. In chorioamnionitis, diminished IL-10 levels may provide an ineffective counter regulatory response to elevated maternal prostaglandin stimulated by the pro-inflammatory cytokines IL-1, IL-6 and TNF. Elevated levels of these pro-inflammatory cytokines in utero may serve as a maternal [28] and fetal [29] signal for the onset of premature parturition. The higher proportion of early PTB in women with the *IL10 ATA *haplotype may also be a reflection of failure of tocolysis in the presence of infection [26]. Our finding that the carriage of the *IL10 ATA *haplotype is more common in women with histologic chorioamnionitis suggests that this haplotype may be important in the pathogenesis of histologic chorioamnionitis and subsequent PTB.

MBL is an important innate defense molecule active against a broad range of bacterial, viral, fungal and protozoan pathogens [30]. The *MBL2 *variant alleles in exon 1 correlate with decreased circulating MBL. The single nucleotide substitution of an adenine for a guanine in codon 54 results in replacement of aspartic acid for a glycine in the MBL [31]. Functional MBL is multimeric, consisting of tetramers, pentamers and hexamers of triplets of the basic MBL peptide. This higher order structure is necessary for high avidity interactions between MBL carbohydrate recognition domains and repeated oligosaccharide moieties on pathogens. The MBL codon 54Asp 'B' allele disrupts assembly of *MBL2 *peptide trimers resulting in lower stability and serum levels [32]. Low serum levels of MBL and *MBL2 *genetic variants are associated with infection in a range of contexts including recurrent infection in children and adults [6,14], recurrent vulvovaginal candidiasis [33], recurrent miscarriage [34] and autoimmunity in common variable immunodeficiency disease [18]. The association between *MBL2 *codon 54Asp and histologic chorioamnionitis suggests that this allele may result in low MBL levels, impaired opsonisation and clearance of pathogens, thus facilitating the development of chorioamnionitis.

Fas protein is expressed on the surface of many cell types, such as lymphocytes, epithelial, fibroblasts and certain endothelial cells, and cytokines [7] and hormones [35] found in the placental microenvironment may modulate the immune response by regulating the expression of Fas and Fas ligand. In this study two polymorphisms in the Fas (*TNFRSF6*) gene promoter were studied at positions -1377 and -670, and the -1377A/-670G (*AG*) haplotype was negatively associated with histologic chorioamnionitis. The -1377G/A is situated at the transcription factor SP-1 binding site [22], and the -670A/G substitution is located in the enhancer region and abolishes the binding site of the nuclear transcription element GAS (Gamma Interferon Activation Sequence). The functional significance of these polymorphisms has not been fully elucidated but they may modulate *TNFRSF6 *transcription, Fas expression and thus apoptotic and inflammatory responses. Recent evidence suggests that cells are more sensitive to Fas-mediated apoptosis when levels of Fas expression increase [36]. Apoptotic cell death can modulate host containment of pathogens [19] and thus genetically determined variation in Fas-mediated cell death may influence histologic chorioamnionitis.

Reported studies on gene polymorphisms and adverse pregnancy outcome have mostly examined SNPs in the genes encoding the pro-inflammatory cytokines TNF and IL-1. The *TNF *promoter -308A allele was associated with clinical chorioamnionitis in a small number of women [12]. The *in vitro *functional data of the effects of the *TNF *SNPs are conflicting and may be context dependent and influenced by strong linkage disequilibrium with other intra-and-extra-genic polymorphisms (e.g. with lymphotoxin alpha and classical HLA genes [37], not examined in this study). In contrast, in this study the *TNF*-308A could not be identified as a susceptibility or severity factor for chorioamnionitis.

SNPs of the IL1 cluster have been examined from different ethnic groups, in particular *IL1B *and *IL1RN *in women with a history of recurrent pregnancy loss [38], PTB [39], and in *in vitro *studies[11]. We did not find associations between genes of the IL1 cluster or *IL1R1 *and histologic chorioamnionitis and this may partly be explained by differences in linkage disequilibrium patterns across different ethnic populations that can lead to different association results [40].

Our data support the role of *IL10*, *MBL2 *and *TNFRSF6 *variants in determining the risk of histologic chorioamnionitis. However there are limitations to the study, such as small sample size. The range and number of gene polymorphisms examined and analysed by univariate and multiple logistic regression in this study is notable, but some correction for multiple analyses is required [41]. The appropriate method still remains a contentious issue. The Bonferroni method was not conducted, as this is a conservative method and leads to overcorrection [42]. Also our study is a hypothesis-generating exploratory study. SNP frequencies in women with and without chorioamnionitis are available to be included in meta-analysis to confirm or reject these findings (Additional file 1).

## Conclusion

In conclusion, the frequency differences in this study highlight the significance and biological relevance of genetic factors, IL-10, MBL and Fas regulation among women with acute chorioamnionitis and adverse pregnancy outcome.

## Competing interests

The author(s) declare that they have no competing interests.

## Authors' contributions

**MA **contributed to study design, recruited study subjects, abstracted medical records, entered data, performed preliminary statistical analyses and drafted the manuscript.

**PH **provided design regarding immunoregulatory genes, interpretation of results and preparation of manuscript.

**CM **conceived methodology and supervision of PCR testing, interpretation of test results and preparation of manuscript.

**SH **supervised preparation of DNA and performed all PCR tests.

**JR **provided clinical authorisation and support for the study. Provided input regarding clinical database design, and provided critical analysis for obstetric correctness.

**HM **conceived and coordinated the study, conceived medical record review, reviewed statistical analyses and preparation of manuscript.

All authors read and approved the final manuscript.

## Pre-publication history

The pre-publication history for this paper can be accessed here:



## Supplementary Material

Additional File 1 Gene alleles, haplotypes and genotypes in Caucasoid Australian women with preterm birth before 35 weeks gestation and histologic chorioamnionitis. Frequency distribution of immunoregulatory gene alleles, haplotypes and genotypes in Caucasoid Australian women with preterm birth before 35 weeks gestation stratified by histologic chorioamnionitis. Click here for file
