# Supplementary material for: Polymorphisms in immunoregulatory genes and the risk of histologic chorioamnionitis in Caucasoid women: a case control study
Source: BMC Pregnancy Childbirth. 2005 Feb 21;5:4. doi: 10.1186/1471-2393-5-4 (PMC554771; doi:10.1186/1471-2393-5-4)
Supplement: Additional File 1 — Gene alleles, haplotypes and genotypes in Caucasoid Australian women with preterm birth before 35 weeks gestation and histologic chorioamnionitis. Frequency distribution of immunoregulatory gene alleles, haplotypes and genotypes in Caucasoid Australian women with preterm birth before 35 weeks gestation stratified by histologic chorioamnionitis. [file 1471-2393-5-4-S1.doc]

Additional file 1: Gene alleles, haplotypes and genotypes in Caucasoid Australian women with preterm birth before 35 weeks gestation and histologic chorioamnionitis.

| **Gene** | **Variant** | **Histologic** | **No** | **Univariate** |
| --- | --- | --- | --- | --- |
|  |  | **Chorio-** | **Histologic** | **analysis** |
|  |  | **amnionitis** | **Chorio-** | **OR, 95% CI,** |
|  |  |  | **amnionitis** | **uncorrected *P*** |
|  |  |  |  | **value** |
| TGFB1 | Allele | **n = 138** | **n = 224** |  |
|  | -800 G | 121 (0.88) | 206 (0.92) | ns |
|  | -800 A | 17 (0.12) | 18 (0.08) | ns |
|  | -509 C | 105 (0.76) | 155 (0.69) | ns |
|  | -509 T | 33 (0.24) | 69 (0.31) | ns |
|  | Haplotypes | **n = 138** | **n = 224** |  |
|  | -800/-509 |  |  |  |
|  | GC | 88 (0.64) | 137 (0.61) | ns |
|  | GT | 33 (0.24) | 69 (0.31) | ns |
|  | AC | 17 (0.12) | 18 (0.08) | ns |
|  | AT | 0 (0.00) | 0 (0.00) | ns |
|  |  | **n = 69** | **n = 112** |  |
|  | GC/AC | 6 (0.09) | 12 (0.11) | ns |
|  | AC/AC | 2 (0.03) | 1 (0.01) | ns |
|  | GC/GT | 22 (0.32) | 37 (0.33) | ns |
|  | GT/GT | 2 (0.03) | 14 (0.13) | 0.2 (0.02-1.0), 0.03 |
|  | GC/GC | 30 (0.43) | 44 (0.39) | ns |
|  | GT/AC | 7 (0.10) | 4 (0.04) | ns |
|  | Genotypes |  |  |  |
|  | -800 G/G | 54 (0.78) | 95 (0.85) | ns |
|  | -800 G/A | 13 (0.19) | 16 (0.14) | ns |
|  | -800 A/A | 2 (0.03) | 1 (0.01) | ns |
|  | -509 C/C | 38 (0.55) | 57 (0.51) | ns |
|  | -509 C/T | 29 (0.42) | 41 (0.37) | ns |
|  | -509 T/T | 2 (0.03) | 14 (0.13) | 0.2 (0.02-0.96), 0.03 |
|  |  |  |  |  |
| ***IL4*** | Allele | **n = 138** | **n = 224** |  |
|  | -590 C | 125 (0.91) | 198 (0.88) | ns |
|  | -590 T | 13 (0.09) | 26 (0.12) | ns |
|  | **Genotype** | **n = 69** | **n = 112** |  |
|  | -590 C/C | 58 (0.84) | 88 (0.79) | ns |
|  | -590 C/T | 9 (0.13) | 22 (0.20) | ns |
|  | -590 T/T | 2 (0.03) | 2 (0.02) | ns |
|  |  |  |  |  |
| IL10 | Allele | **n = 138** | **n = 224** |  |
|  | -1082 A | 76 (0.55) | 101 (0.45) | 1.5 (1.0-2.3), 0.06 |
|  | -1082 G | 62 (0.45) | 123 (0.55) | 0.7 (0.4-1.1), 0.07 |
|  | -819 C | 98 (0.71) | 178 (0.79 | 0.6 (0.4-1.1), 0.07 |
|  | -819 T | 40 (0.29) | 46 (0.21) | 1.6 (0.9-2.7), 0.07 |
|  | -592 C | 98 (0.71) | 178 (0.79) | 0.6 (0.4-1.1), 0.07 |
|  | -592 A | 40 (0.29) | 46 (0.21) | 1.6 (0.9-2.7), 0.07 |
|  | Haplotypes |  |  |  |
|  | -1082/-819/592 | |  |  |
|  | ACC | 36 (0.26) | 55 (0.25) | ns |
|  | ATA | 40 (0.29) | 46 (0.21) | 1.6 (0.9-2.7), 0.07 |
|  | GCC | 62 (0.45) | 123 ( 0.55) | 0.7 ( 0.4-1.1), 0.06 |
|  |  | **n = 69** | **n = 112** |  |
|  | ACC/ACC | 6 (0.09) | 9 (0.08) | ns |
|  | ATA/ATA | 6 (0.09) | 9 (0.08) | ns |
|  | GCC/GCC | 16 (0.23) | 38 (0.34) | 0.6 (0.3-1.2), 0.13 |
|  | ATA/GCC | 17 (0.25) | 19 (0.17) | ns |
|  | ACC/GCC | 13 (0.19) | 28 (0.25) | ns |
|  | ACC/ATA | 11 (0.16) | 9 (0.08) | 2.3 (0.8-6.3), 0.10 |
|  | Genotypes |  |  |  |
|  | -1082 A/A | 23 (0.33) | 27 (0.24) | ns |
|  | -1082 A/G | 30 (0.44) | 47 (0.42) | ns |
|  | -1082 G/G | 16 (0.23) | 38 (0.34) | 0.6 (0.3-1.2), 0.13 |
|  | -819 C/C | 35 (0.51) | 75 (0.67) | 0.5 (0.3-1.0), 0.03 |
|  | -819 C/T | 28 (0.41) | 28 (0.25) | 2.1 (1.0-4.1), 0.03 |
|  | -819 T/T | 6 (0.09) | 9 (0.08) | ns |
|  | -592 C/C | 35 (0.51) | 75 (0.67) | 0.5 (0.3-1.0), 0.03 |
|  | -592 A/C | 28 (0.41) | 28 (0.25) | 2.1 (1.0-4.1), 0.03 |
|  | -592 A/A | 6 (0.09) | 9 (0.08) | ns |
|  |  |  |  |  |
| IL1RN | Allele | **n = 138** | **n = 224** |  |
|  | +11100 C | 38 (0.28) | 67 (0.30) | ns |
|  | +11100 T | 100 (0.72) | 157 (0.70) | ns |
|  | **Genotype** | **n = 69** | **n = 112** |  |
|  | +11100 C/C | 5 (0.07) | 12 (0.11) | ns |
|  | +11100 C/T | 28 (0.41) | 43 (0.38) | ns |
|  | +11100 T/T | 36 (0.52) | 57 (0.51) | ns |
|  |  |  |  |  |
| IL1A | Allele | **n = 138** | **n = 224** |  |
|  | -889 T | 31 (0.22) | 59 (0.26) | ns |
|  | -889 C | 107 (0.78) | 165 (0.74) | ns |
|  | **Genotype** | **n = 69** | **n = 112** |  |
|  | -889 C/C | 42 (0.61) | 63 (0.56) | ns |
|  | -889 C/T | 23 (0.33) | 39 (0.35) | ns |
|  | -889 T/T | 4 (0.06) | 10 (0.09) | ns |
|  |  |  |  |  |
| IL1B | Allele | **n = 138** | **n = 224** |  |
|  | +3962 G | 111 (0.80) | 174 (0.78) | ns |
|  | +3962 A | 27 (0.20) | 50 (0.22) | ns |
|  | **Genotype** | **n = 69** | **n = 112** |  |
|  | +3962 G/G | 44 (0.64) | 68 (0.61) | ns |
|  | +3962 G/A | 23 (0.33) | 38 (0.34) | ns |
|  | +3962 A/A | 2 (0.03) | 6 (0.05) | ns |
|  |  |  |  |  |
|  | Allele | **n = 138** | **n = 224** |  |
|  | -511 T | 44 (0.32) | 69 (0.31) | ns |
|  | -511 C | 94 (0.68) | 155 (0.69) | ns |
|  | **Genotype** | **n = 69** | **n = 112** |  |
|  | -511 T/T | 8 (0.12) | 12 (0.11) | ns |
|  | -511 T/C | 28 (0.41) | 45 (0.40) | ns |
|  | -511 C/C | 33 (0.48) | 55 (0.49) | ns |
|  |  |  |  |  |
| IL6 | Allele | **n = 138** | **n = 224** |  |
|  | -174 C | 55 (0.40) | 91 (0.41) | ns |
|  | -174 G | 83 (0.60) | 133 (0.59) | ns |
|  | **Genotypes** | **n = 69** | **n = 112** |  |
|  | -174 C/C | 12 (0.17) | 15 (0.13) | ns |
|  | -174 G/C | 31 (0.45) | 61 (0.55) | ns |
|  | -174 G/G | 26 (0.38) | 36 (0.32) | ns |
|  |  |  |  |  |
| IL1R1 | Allele | **n = 138** | **n = 224** |  |
|  | +970 C | 95 (0.69) | 143 (0.64) | ns |
|  | +970 T | 43 (0.31) | 81 (0.36) | ns |
|  | **Genotype** | **n = 69** | **n = 112** |  |
|  | +970 C/C | 30 (0.43) | 47 (0.42) | ns |
|  | +970 C/T | 35 (0.51) | 49 (0.44) | ns |
|  | +970 T/T | 4 (0.06) | 16 (0.14) | 0.4 (0.1-1-2), 0.08 |
|  |  |  |  |  |
| TNF | Allele | **n = 138** | **n = 224** |  |
|  | +488 G | 121 (0.88) | 205 (0.92) | ns |
|  | +488 A | 17 (0.12) | 19 (0.08) | ns |
|  | -238 G | 129 (0.93) | 214 (0.96) | ns |
|  | -238 A | 9 (0.07) | 10 (0.04) | ns |
|  | -308 G | 118 (0.86) | 179 (0.80) | ns |
|  | -308 A | 20 (0.14) | 45 (0.20) | ns |
|  | **Haplotype** |  |  |  |
|  | +488/-238/-308 | |  |  |
|  | GGG | 92 (0.67) | 150 (0.67) | ns |
|  | GGA | 20 (0.14) | 45 (0.20) | ns |
|  | GAG | 9 (0.07) | 10 (0.04) | ns |
|  | AGG | 17 (0.12) | 19 (0.08) | ns |
|  |  | **n = 69** | **n = 112** |  |
|  | GGG/GGG | 28 (0.41) | 50 (0.45) | ns |
|  | GGA/GGA | 1 (0.01) | 4 (0.04) | ns |
|  | GAG/AGG | 1 (0.01) | 1 (0.01) | ns |
|  | AGG/AGG | 0 (0.00) | 1 (0.01) | ns |
|  | GGG/GAG | 6 (0.09) | 4 (0.04) | ns |
|  | GGA/AGG | 1 (0.01) | 1 (0.01) | ns |
|  | GGG/AGG | 15 (0.22) | 15 (0.13) | 1.8 (0.8-2.3), 0.14 |
|  | GGA/GAG | 2 (0.03) | 5 (0.04) | ns |
|  | GGG/GGA | 15 (0.22) | 31 (0.28) | ns |
|  | Genotype |  |  |  |
|  | +488 G/G | 52 (0.75) | 94 (0.84) | ns |
|  | +488 G/A | 17 (0.25) | 17 (0.15) | 1.8 ( 0.8-4.2), 0.11 |
|  | +488 A/A | 0 (0.00) | 1 (0.01) | ns |
|  | -238 G/G | 60 (0.87) | 102 (0.91) | ns |
|  | -238 G/A | 9 (0.13) | 10 (0.09) | ns |
|  | -238 A/A | 0 (0.00) | 0 (0.00) | ns |
|  | -308 G/G | 50 (0.73) | 71 (0.63) | ns |
|  | -308 G/A | 18 (0.26) | 37 (0.33) | ns |
|  | -308 A/A | 1 (0.01) | 4 (0.04) | ns |
|  |  |  |  |  |
| TNFRSF6 | |  |  |  |
|  | Allele | **n = 138** | **n = 224** |  |
|  | -1377 A | 10 (0.07) | 33 (0.15) | 0.5 ( 0.2-1.0), 0.03 |
|  | -1377 G | 128 (0.93) | 191 (0.85) | 2.2 ( 1.0-5.2), 0.03 |
|  | -670 A | 80 (0.58) | 118 (0.53) | ns |
|  | -670 G | 58 (0.42) | 106 (0.47) | ns |
|  | **Haplotype** |  |  |  |
|  | -1377/-670 |  |  |  |
|  | GG | 48 (0.35) | 73 (0.30) | ns |
|  | AG | 10 (0.07) | 33 (0.15) | 0.4 (0.2-1.0), 0.03 |
|  | GA | 80 (0.58) | 118 (0.53) | ns |
|  | AA | 0 (0.00) | 0 (0.00) | ns |
|  |  | **n = 69** | **n = 112** |  |
|  | GG/GG | 5 (0.07) | 12 (0.11) | ns |
|  | AG/AG | 1 (0.02) | 3 (0.03) | ns |
|  | GA/GA | 22 (0.32) | 34 (0.30) | ns |
|  | AG/GA | 3 (0.04) | 14 (0.13) | 0.3 (0.06-1.2), 0.07 |
|  | GG/AG | 5 (0.07) | 13 (0.12) | ns |
|  | GG/GA | 33 (0.48) | 36 (0.32) | 1.9 (1.0-3.8), 0.03 |
|  | Genotype |  |  |  |
|  | -1377 A/A | 1 (0.01) | 3 (0.03) | ns |
|  | -1377 A/G | 8 (0.12) | 27 (0.24) | 0.4 (0.2-1.0), 0.04 |
|  | -1377 G/G | 60 (0.87) | 82 (0.73) | 2.4 (1.0-6.3), 0.03 |
|  | -670 A/A | 22 (0.32) | 34 (0.30) | ns |
|  | -670 A/G | 36 (0.52) | 50 (0.45) | ns |
|  | -670 G/G | 11 (0.16) | 28 (0.25) | 0.6 (0.2-1.3), 0.15 |
|  |  |  |  |  |
| **MBL2** |  | **n = 138** | **n = 224** |  |
|  | -550 |  |  |  |
|  | H | 56 (0.41) | 79 (0.35) | ns |
|  | L | 82 (0.59) | 145 (0.65) | ns |
|  | -221 |  |  |  |
|  | Y | 109 (0.79) | 163 (0.73) | ns |
|  | X | 29 (0.21) | 61 (0.27) | ns |
|  |  | **n = 69** | **n = 112** |  |
|  | Genotypes |  |  |  |
|  | H/H | 12 (0.17) | 18 (0.16) | ns |
|  | H/L | 32 (0.46) | 43 (0.38) | ns |
|  | L/L | 25 (0.36) | 51 (0.46) | ns |
|  | Genotypes |  |  |  |
|  | Y/Y | 45 (0.65) | 61 (0.54) | 1.6 (0.8-3.1), 0.15 |
|  | Y/X | 19 (0.28) | 41 (0.37) | ns |
|  | X/X | 5 (0.07) | 10 0.09) | ns |
|  | **Codons** | **n = 138** | **n = 224** |  |
|  | A | 106 (0.77) | 174 (0.78) | ns |
|  | Variant |  |  |  |
|  | 52Cys (D) | 5 (0.04) | 18 (0.08) | ns |
|  | 54Asp (B) | 27 (0.20) | 29 (0.13) | 1.6 (0.9-3.0), 0.09 |
|  | 57Glu (C) | 0 (0.00) | 3 (0.01) | ns |
|  | Heterozygous and homozygous promoter haplotypes | | |  |
|  | with a normal coding region (A) | |  |  |
|  |  | **n = 69** | **n = 112** |  |
|  | HYA/HYA | 11 (0.16) | 11 (0.10) | ns |
|  | HYA/LXA | 5 (0.07) | 19 (0.17) | 0.4 (0.1-1.1), 0.06 |
|  | HYA/LYA | 12 (0.17) | 8 (0.07) | 2.7 (1.0-8.2), 0.03 |
|  | LYA/LYA | 2 (0.03) | 11 (0.10) | 0.3 (0.03-1.3), 0.08 |
|  | LXA/LXA | 5 (0.07) | 10 (0.09) | ns |
|  | LXA/LYA | 3 (0.04) | 7 (0.06) | ns |
|  | Heterozygous promoter and variant | |  |  |
|  | coding region haplotypes | |  |  |
|  | HYA/LYB | 11 (0.16) | 5 (0.04) | 4.1 (1.2-16.0), 0.01 |
|  | HYA/HYD | 1 (0.01) | 7 (0.06) | ns |
|  | LYA/LYB | 4 (0.06) | 10 (0.09) | ns |
|  | LYA/LYC | 0 (0.00) | 2 (0.02) | ns |
|  | LYA/HYD | 3 (0.04) | 3 (0.03) | ns |
|  | LXA/LYB | 11 (0.16) | 10 (0.09) | 1.9 (0.7-5.4), 0.15 |
|  | LXA/HYD | 0 (0.00) | 5 (0.04) | ns |
|  | Compound heterozygous coding region variants | | |  |
|  | LYB/LYB | 0 (0.00) | 1 (0.01) | ns |
|  | HYD/LYB | 1 (0.01) | 2 (0.02) | ns |
|  | HYD/ LYC | 0 (0.00) | 1 (0.01) | ns |

**Additional file 1 Footnote:**

Data are presented as allele frequencies (proportion of positive chromosomes), genotype frequencies (heterozygous or homozygous combinations of variants), and haplotype frequencies (ordered combination of alleles on a single chromosome).

Univariate analyses were tested using Chi square analysis of 2 x 2 table, and uncorrected P values, Odds Ratio (OR), and 95% Confidence Intervals (CI). P values < 0.15 are presented.

*MBL2* (mannose-binding lectin) promoter polymorphisms are located at nucleotides –550 G/C (called H/L alleles) and -221 C/G (X/Y alleles). The three single nucleotide polymorphisms in exon 1 are at codon 52 (Arg/Cys) called allele ‘D’, codon 54 (Gly/Asp) allele ‘B’, and codon 57 (Gly/Glu) allele ‘C’. 52Cys, 54Asp and 57Glu are commonly referred to as mutations as they disrupt the assembly of the MBL multimer, resulting in low blood levels. A *MBL2* coding region with no mutations is referred to as ‘A’. The listed *MBL2* haplotypes are associated with different blood levels. The promoter polymorphisms are in complete linkage disequilibrium with the coding variants: the –550/-221 haplotype HY is always on the same chromosome with 52Cys, and the LY with 54Asp and 57Glu. On the same chromosome with a “normal” coding region (‘A”), the promoter haplotypes HY, LY and LX are associated with high, intermediate and low blood levels of multimeric MBL, respectively.

*TNFRSF6* = gene symbol for Fas,

ns, not significant.

Presence of a haplotype (proportion of individuals possessing at least one copy of a haplotype) can be deduced from the haplotype/genotype frequencies.
